# Uncovering the Anticancer Potential of Polydatin: A Mechanistic Insight

**DOI:** 10.3390/molecules27217175

**Published:** 2022-10-23

**Authors:** Muhammad Ajmal Shah, Ayesha Hamid, Hafiza Ishmal Faheem, Azhar Rasul, Tourki A. S. Baokbah, Muhammad Haris, Rimsha Yousaf, Uzma Saleem, Shabnoor Iqbal, Maria Silvana Alves, Zahid Khan, Ghulam Hussain, Ifat Alsharfi, Haroon Khan, Philippe Jeandet

**Affiliations:** 1 Department of Pharmacy, Hazara University, Mansehra 21300, Pakistan; 2 Faculty of Pharmaceutical Sciences, Government College University, Faisalabad 38000, Pakistan; 3 Department of Zoology, Faculty of Life Sciences, Government College University, Faisalabad 38000, Pakistan; 4 Department of Medical Emergency Services, College of Health Sciences-AlQunfudah, Umm Al-Qura University, Makkah 21955, Saudi Arabia; 5 Faculty of Pharmaceutical Sciences, Universiteit Gent, Ghent 9000, Belgium; 6 Laboratory of Cellular and Molecular Bioactivity, Department of Pharmaceutical Sciences, Faculty of Pharmacy, Federal University of Juiz de Fora, Juiz de Fora 36036-900, Brazil; 7 Department of Pharmacognosy, Faculty of Pharmacy, Federal Urdu University of Arts, Science & Technology, Karachi 75300, Pakistan; 8 Department of Physiology, Faculty of Life Sciences, Government College University, Faisalabad 38000, Pakistan; 9 Department of Biology, Jamoum University College, Umm Al-Qura University, Makkah 21955, Saudi Arabia; 10 Department of Pharmacy, Abdul Wali Khan University Mardan, Mardan 23200, Pakistan; 11 Research Unit Induced Resistance and Plant Bioprotection, University of Reims Champagne-Ardenne, USC INRAe 1488, 51100 Reims, France

**Keywords:** phenol compounds, polydatin, 3-*O*-β-d-resveratrol-glucopyranoside, breast cancer, cervical cancer, lung cancer, ovarian cancer

## Abstract

Polydatin or 3-*O*-β-d-resveratrol-glucopyranoside (PD), a stilbenoid component of *Polygonum cuspicadum* (Polygonaceae), has a variety of biological roles. In traditional Chinese medicine, *P. cuspicadum* extracts are used for the treatment of infections, inflammation, and cardiovascular disorders. Polydatin possesses a broad range of biological activities including antioxidant, anti-inflammatory, anticancer, and hepatoprotective, neuroprotective, and immunostimulatory effects. Currently, a major proportion of the population is victimized with cervical lung cancer, ovarian cancer and breast cancer. PD has been recognized as a potent anticancer agent. PD could effectively inhibit the migration and proliferation of ovarian cancer cells, as well as the expression of the PI3K protein. The malignancy of lung cancer cells was reduced after PD treatments via targeting caspase 3, arresting cancer cells at the S phase and inhibiting NLRP3 inflammasome by downregulation of the NF-κB pathway. This ceases cell cycle, inhibits VEGF, and counteracts ROS in breast cancer. It also prevents cervical cancer by regulating epithelial-to-mesenchymal transition (EMT), apoptosis, and the C-Myc gene. The objective of this review is thus to unveil the polydatin anticancer potential for the treatment of various tumors, as well as to examine the mechanisms of action of this compound.

## 1. Introduction

Cancer, a multifactorial disease, is a rapidly growing condition in which cells grow abnormally and invade many parts of the body, showing a metastasis behavior. There are many types of cancer known so far [1]. Cancer of the breast, cervix, lungs, and ovaries are the most prevalent types of the disease. About 2.2 million new instances of lung cancer, 2.3 million new cases of breast cancer, and 0.6 million new cases of cervical cancer were detected globally in 2020. The number of new cases of ovarian cancer in 2018 was close to 0.3 million [2,3,4,5]. Totally, 10 million deaths have been estimated in 2020 by cancer and it has become the most prominent cause of mortality. Breast cancer and lung cancer are the leading types of cancer, with increased cases worldwide. Treatment strategies include anticancer drugs, chemotherapy, immunotherapy, and hormonal treatments [6]. Various types of cancers respond to conventional drug therapies such as alkylating drugs, intercalating agents, topoisomerase inhibitors, antimitotic drugs, and antimetabolites as well as kinase inhibitors, but mutations assist the cell to develop resistance. Targeted chemotherapy is effective in some malignancies, but the side effects on normal cells and its high cost have limited its use. Immunotherapy and targeted monoclonal antibodies have also been recognized as successful approaches against specific cancers, but a restricted number of cancers can be totally treated using these curative methods. Unluckily, resistance to cancer therapies, side effects, and high cost continue to be challenging and increase the rate of increased mortality [7]. 

On the other hand, plants have been employed since ancient times for the extraction of their valuable bioactive compounds to promote the health and well-being of people [8]. Despite the popup of synthetic therapeutic molecules, natural products are still in use for the mitigation and prevention of diseases [9]. Research is developing a means to find out phytochemicals from natural sources by utilizing different approaches [10]. Polydatin (PD), a compound belonging to the stilbene family [11,12,13], is extracted from the roots of *Polygonum cuspidatum* (Polygonaceae). PD is very famous in China because of its usage as a painkiller and febrifuge. The trans form of PD is well known for its high therapeutic potential [10]. The anticancer activity of PD is mediated by several mechanisms such as control of reactive oxygen species (ROS) [14] and suppression of the PI3K/AKT pathway [15]. In several investigations, PI3K/AKT inhibitors were found to improve the treatment effectiveness of 2-deoxy-D-glucose (2-DG) and PD [16,17]. Furthermore, PD has attracted a lot of attention because of its positive impact on glucose and lipid management [18]. PD has therefore enhanced 2-DG’s anticancer effects via regulating glucose metabolism, blocking the PI3K/AKT, or through other pathways. PD has been investigated to reduce the growth of HeLa cells by causing these cells to enter the S phase, promote cell death, and lower AKT, mTOR PI3K, mRNA expression levels. It was also discovered that PD may limit cervical cancer HeLa cell proliferation and induce apoptosis, and the process could be linked to blockage of the PI3K/AKT/mTOR signaling pathway and gene downstream expression [15]. The anticancer potential of PD has been observed by many researchers by using different cell lines like liver, cervical, and nasopharyngeal cancer cell lines [10]. The objective of this review is thus to unveil PD anticancer potential for the treatment of various tumors, as well as to examine the mechanisms of action of this molecule.

## 2. Polydatin Chemistry and Biosynthesis

**Figure 1:**
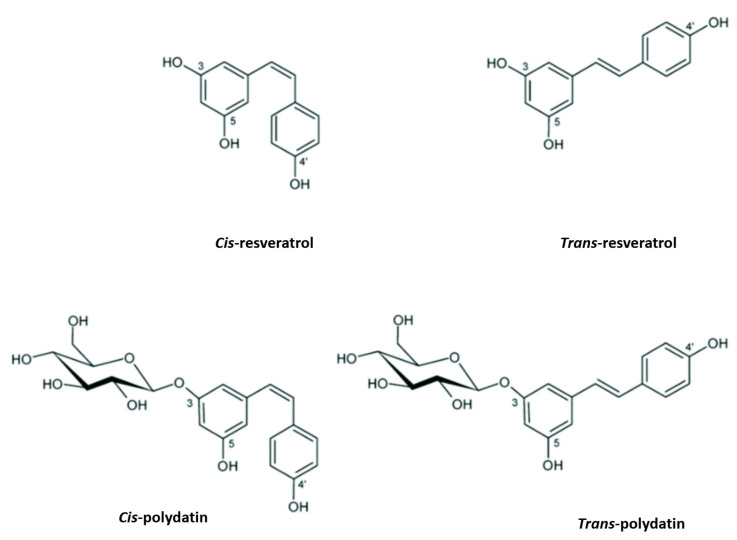
Resveratrol and polydatin isomers, trans and cis.

PD, also known as piceid (3-*O*-β-d-resveratrol glucopyranoside), (*E*)-polydatin, transpolydatin, (*E*)-piceid, is a monocrystalline substance that was first isolated from the roots and rhizome of *P. cuspidatum* Sieb. It is a stilbene derivative of the phytoalexin resveratrol (3,4′,5-trihydroxystilbene), in which the glucoside group linked to position C-3 replaces the hydroxyl group (Figure 1). The transisomers of stilbenes generally display a higher bioactivity than their cisisomer counterparts (Figure 1) [19]. Previously, scientists scrutinized this compound for its ability to help with heart- and liver-associated disorders [20,21]. 

The polyketide and phenylpropanoid routes are used to form PD. The first step in the production of PD is the deamination of phenylalanine by phenylalanine ammonia lyase (PAL), which affords cinnamic acid. Cinnamate-4-hydroxylase (C4H) subsequently hydroxylates cinnamic acid to produce *p*-coumaric acid. Coenzyme A (CoA) ligation then takes place through *p*-coumaroyl-CoA ligase activity. Finally, *p*-coumaroyl-CoA and three molecules of malonyl-CoA are combined together by stilbene synthase (STS) to produce resveratrol [22]. Transresveratrol can then be further metabolized to form additional stilbenoids, like polydatin, by the action of glucosyltransferases on resveratrol [23]. Interestingly, PD has also been produced on a small scale through microbial resveratrol transformation by the *Bacillus cereus* strain UI 1477 [24]. American pokeweed (*Phytolacca americana* L., Phytolaccaceae) cell suspension cultures have the ability to glucosylate transresveratrol and produce PD, as well [25]. An engineered *Escherichia coli* strain harboring tyrosine ammonia lyase, cinnamoyl/*p*-coumaroyl-coenzyme A ligase and stilbene synthase genes was used to produce PD [26].

The most common dietary sources of PD are peanuts, dairy products, chocolate, and grapes [27,28]. The greatest PD concentrations were found in cocoa powder (7.14 µg/g), followed by semisweet chocolate baking chips (2.01 µg/g), dark chocolates (1.8 µg/g), milk chocolates (0.44 µg/g), and chocolate syrups (0.35 µg/g). Nevertheless, red wine may contain as much as 29.2 mg/L of PD [19]. The highest concentration of PD in mulberry roots was 3.15 µg/g. fresh weight [29]. Since the glucoside content of PD typically exceeds that of the aglycone in red wine and other grape products, it has attracted a lot of interest, much more than resveratrol. The precise wine proportions of glycosylated to aglycone forms are affected by a variety of variables, including fermentation techniques and environmental conditions in the vineyards. Transresveratrol is found in red wine at concentrations of up to 14.3 mg/L and PD in concentrations of up to 29.2 mg/L, or about equal molar levels (60–70 µM) of the aglycone and the glucosylated form; white wine has 100 times less PD than red wine does [30]. In elaborating white wine, just the juice is fermented, whereas in making red wine, both the skins and the seeds are left on the juices until after fermentation is completed, leading to greater concentrations in the final product. Red grape skins typically have a higher PD content than white grape skins, but this can vary widely (from 50 to 200 mg/kg dry weight) across different varieties and vintages of the same variety of grape [31]. Spectrum analysis of eluting peaks from a HPLC system was used to determine the amounts of transpiceid, cispiceid, transresveratrol, and cisresveratrol in 36 different sorts of grape juices. Grape juices mostly included polydatin. The average levels of transpiceid, cispiceid, transresveratrol, and cisresveratrol in red grape juices were 3.38 mg/L, 0.79 mg/L, 0.50 mg/L, and 0.06 mg/L, respectively [32].

The Chinese resident meals guide recommends that adults consume 500 g of vegetables and 200 to 400 g of fruits daily; if we assume according to this recommendation by eating 200 g of celery, 100 g of chili pepper, 200 g of edible amaranth or leaf lettuce, 10 g of black soya beans, and one apple (200 g), then our daily PD intake would range from 100 to 1700 mg. The mulberry may increase our daily PD consumption to 3700 mg. The effective dose of PD intake can be around 2500–5000 mg/day for a 50 kg adult [33].

**Figure 2:**
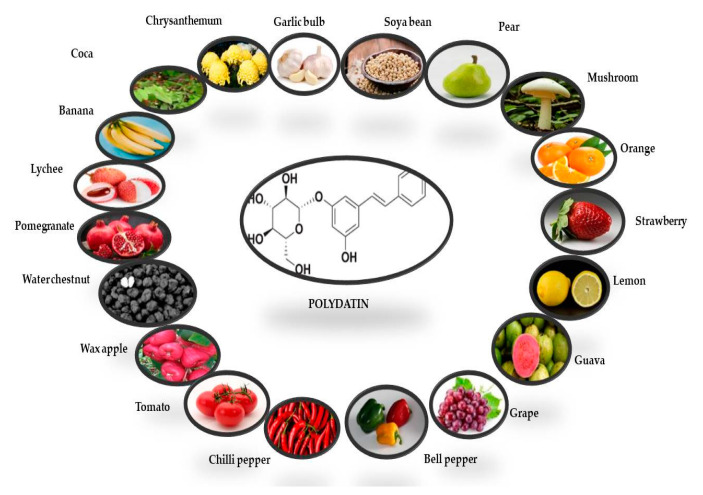
Potential dietary sources of polydatin derivative (transisomer). The quantities of PD in each fruit and vegetable have been derived from Peng et al. [35].

The presence of PD was first reported in the grape skin. Red, white, and grape juices are the main sources of PD, while *rosé* and effervescent wines mostly contain cisPD. Transresveratrol is more prevalent in grapes, berries, peanuts, and pistachios [34]. PD can also be found in a variety of fruit and vegetable foods, beer, cocoa-containing goods, and chocolate products (Figure 2) [35,36]

The primary source of PD is the roots and rhizomes of *Fallopia japonica*, which have a long history of use in traditional Chinese and Japanese medicines such as analgesics, antipyretics, diuretics, anticancers and expectorants, as well as in the treatment of atherosclerosis [37]. However, this compound is found in a number of other taxa, including *Rosa*, *Rumex*, *Picea*, *Malus*, and species of *Quercus* [38]. Peng et al. [35] used chromatographic techniques to quantify polydatin in fruits and vegetables. The polydatin contents of some vegetables and fruits are summarized in Table 1.

## 3. Role of Polydatin in Cancer

PD partly exerts its anticancer activity by enhancing antioxidant activity. PD, like other polyphenols, carries out strong antioxidant activity by neutralizing ROS and boosting the body’s natural antioxidant defences. Its chemical structure of a long-conjugated system confers the compound its substantial antioxidant effects. The resistance of PD to enzymatic oxidation was found to be higher than that of resveratrol. It appears that many of the polydatin biological actions are mediated by antioxidant pathways. In vitro, PD displayed IC_50_ values of 87, 20, and 125 μg/mL for scavenging of the free radicals ABTS (2,2′-Azino-bis (3-ethylbenzthiazoline-6-sulfonic acid), and DPPH (2,2-Diphenyl-1-(2,4,6-trinitrophenyl)-hydrazyl), and •O_2_, respectively [41]. The scavenging effect of PD increases in a dose-dependent manner (0.05–2 μM) in the phenanthroline-Fe^2+^ system, and PD was shown to exhibit a scavenging activity of hydroxyl radicals more effective than those of resveratrol or vitamin C [42].

The earlier investigations from Yousef et al. [43] have reported that PD reduces ROS generation to protect the cell from oxidative stress. Therefore, oxidative stress was induced with H_2_O_2_ in RINm5F cells, and these latter were treated either with or without PD (20 and 40 μg/mL, 24 h), intracellular ROS being evaluated by the dichloro-fluorescein (DCF) assay. The mean fluorescence intensity (MFI) of cells treated with H_2_O_2_ was significantly higher compared to cells treated with a negative control dye, suggesting a buildup of ROS. Treatment with PD (40 g/mL) effectively mitigated the formation of ROS due to PD antioxidant properties. [43]. Yousef et al. [43] also depicted pancreatic lipid peroxidation as being significantly reduced in PD-treated diabetic rats as a consequence of an increase in the antioxidant enzymatic activity of catalase (CAT), superoxide dismutase (SOD), and GPx, following oral therapy with PD.

Otherwise, the anticancer activity of PD on tumor growth has been extensively studied in several cell culture and animal tumor models. Oncology has now been recognized as the most important area of concern in the field of cancer research [44]. Various approaches being used currently include chemotherapy, immune therapy, radiotherapy, surgery, drug combination, antibodies, and some others, all of them having their own side effects. Several researchers tried to combine different targeted cancer therapies to increase their effectiveness and more significantly hinder resistance to therapy; unfortunately, clinical trials have not shown satisfactory results [44]. PD has been recognized as a potent anticancer agent, with the ability to regulate various signaling pathways involved in the progression of several kinds of cancers [45]. The mechanisms by which PD acts in cancer include cell cycle regulation, apoptosis, autophagy, signaling pathways, epithelial-to-mesenchymal transition (EMT), inhibition of inflammation and metastasis, and regulation of enzymes related to oxidative stress [46,47,48].

## 4. Anticancer Activity of Polydatin on Liver, Colon, Bone, Breast, Lung, Cervical, and Ovarian Cancer Proliferation

### 4.1. Liver Cancer

Cancers are the top cause of death for people worldwide, especially those who are 55 and older. Chemotherapy is still the best option for many types of cancer when surgery has been exhausted. Hepatocellular carcinoma (HCC), lung cancer, and breast cancer are just a few examples of the many tumors for which promising results have been obtained from the use of natural substances such as potential medications in recent years [49,50]. Primary HCC is a prevalent secondary malignancy in patients with cirrhosis and other chronic liver disorders. Among cancer-related fatalities, HCC ranks third [51]. Unfortunately, advanced HCC cannot be effectively treated with currently available chemotherapeutic drugs [50,52]. To this end, it is important to have more potent chemicals that might lead to new therapies for treating HCC, especially in its later stages. PD exhibited considerable cytotoxicity in a concentration- and time-dependent manner against HCC (hepatocellular carcinoma) cells at 100 µM and 150 µM concentrations, inducing apoptosis and limited G2/M cell cycle arrest while phosphorylated p-signal transducer and activator of transcription 3 (STAT3), p-Janus kinase 1 and (p)-protein kinase B (AKT) were downregulated [53]. PD may also induce apoptosis by increasing the Bax/Bcl-2 ratio and lowering the Wnt/-catenin signaling in SMMC-7721 and HepG2 cells, both of which are used for the modelling of hepatocellular cancer. Cancer metastasis is thought to be facilitated in large part by the invasion and migration of cancer cells. Treatment with PD inhibited the invasion and migration of HCC cells in two different assays: one measuring invasion and the other measuring wound healing [54]. This suggests that PD may be a useful natural small molecule medication for the treatment of liver cancer at an early stage.

### 4.2. Colon Cancer

PD inhibited cell differentiation of CaCo-2 human colon cancer cells through inhibition of Hsp27 and vimentin expression (IC_50_ values of 72 and 192 μM for exponentially developing and postconfluent cells, respectively). After treatment with PD (240 μM), the cell cycle arrested at the G1 phase, coinciding with an increase in the cleaved poly-(ADP-ribose) polymerase. Both the total and phosphorylated versions of Akt were decreased though ERK1/2 phosphorylation and p21 expression, which were both enhanced in the CaCo-2 cell line [55]. The growth inhibition of Caco-2 intestinal epithelial cells exerted by PD was concentration-dependent (1–50 μM) and occurred via cell cycle arrest in the G0/G1 (10–25 μM) and apoptosis induction. Caco-2 cells treated with 50 μM PD displayed DNA fragmentation, whereas those treated with 100 μM resveratrol underwent apoptosis [56]. Use of flow cytometry and immunoblotting in the investigation by Bae et al. [57] showed that apoptosis was triggered by the disruption of calcium regulation and the expression levels of associated proteins in HT-29 and HCT116 cell lines. Both the MAPK and PI3K/AKT signaling pathways were shown to be downregulated by polydatin. It was also demonstrated that the combination of polydatin and 5-fluorouracil (5-FU) was effective in inhibiting drug resistance in 5-FU-resistant cells. Therefore, the results of this study support further research on PD in order to see whether or not it can be developed as a novel therapeutic agent for the treatment of colon cancer [57]. Polydatin significantly reduced cell growth and increased apoptosis in CRC cell lines [58]. It was shown that miR-382 specifically targets PD-L1. PD ability to upregulate miR-382 enables it to inhibit PD-L1 expression. Furthermore, PD regulates miR-382 to reduce CRC tumor development in vivo, where it suppresses tumor formation and induces death of CRC cells [58]. PD suppressed cell growth in RPMI 8226 multiple myeloma cells through the mTOR/p70s6k signaling pathway [58]. The IC_50_ values for PD were 131 μM and 93 μM at 24 and 48 h, respectively in RPMI 8226 cells. At a concentration of 50 μM, PD triggered apoptosis by upregulating caspase-3, caspase-9 and Bax levels and decreasing Bcl-2. The same concentrations also stimulated autophagy by increasing the expression of Beclin 1, Atg5, and LC3II. Phosphorylation of mTOR and p70s6 k was reduced [58].

### 4.3. Bone Cancer

The child population has a higher incidence of osteosarcoma (OS) than any other primary bone tumor [59]. There are around 3.4 new instances per 1,000,000 persons each year [60,61], with men being more affected than women (5.4 per 100,000 vs. 4.0 per 100,000). Although osteosarcoma can affect any part of the skeleton, it most commonly occurs in the long bones (90%) and the knee (50%). Researches have shown that genetic and epigenetic alterations disrupt the normal differentiation process that begins with mesenchymal stem cells, leading to the development of OS [62]. PD-induced apoptosis was triggered by the downregulation of β-catenin signaling and the upregulated expressions of Bax/Bcl-2 and caspase-3 in MG63 and 143B OS cells at dose-dependent concentrations, and a significantly reduced cell growth was observed [63]. The effect of PD on osteosarcoma cells, both before and after radiation therapy, was described [64]. In these experiments, PD was found to reduce bone cancer progression. Polydatin significantly upregulated cell cycle arrest in S-phase and elevated bone alkaline phosphatase activity in vitro. Pretreatment with PD activated the Wnt/β-catenin pathway and enhanced osteogenic marker expression as well as decreasing tumor cell survival, demonstrating a radiosensitizing effect when combined with radiation therapy for OS [64].

### 4.4. Breast Cancer

The class of cancer responsible for the highest mortality in women is breast cancer, (BC)which alone causes 25% of death in women as compared to other types of cancers [65]. The most common treatment used for breast cancer is chemotherapy, besides surgical and hormonal treatment [66]. Many factors including dysregulated autophagy, imbalanced apoptosis, changes in gene levels, and certain molecular signaling pathways are the leading causes of BC. These will be discussed one by one.

**Figure 3:**
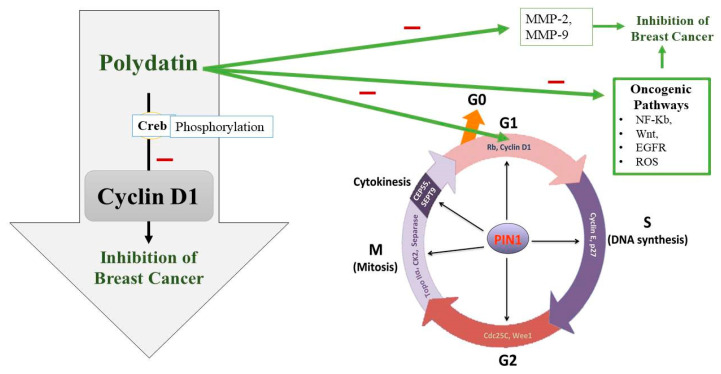
Polydatin (PD) potential to inhibit the G1 phase of the cell cycle along with other oncogenic pathways. PD appears to interfere with Creb phosphorylation, which downregulates Cyclin D1 and thus terminates the cell cycle at G1 phase and in turn inhibits breast cancer growth. PIN1: peptidyl-prolyl cis/trans isomerase, CEP55: Centrosomal protein 55, CK2: casein kinase 2, Rb: the retinoblastoma protein.

Uncontrolled cell division is also seen due to a disturbed cell cycle. The cell cycle consists of four major phases which are controlled by certain cyclin-dependent kinases (CDKs) and their partners, cyclins [67]. Sometimes, these CDKs and cyclins are upregulated or overexpressed leading to BC pathogenesis [68]. Upregulation of CDK2 and overexpression of cyclins E and B1 are observed in BC [69,70]. So, cell cycle arrest can be targeted for preventing BC progression. A transcription factor called Creb, which regulates many genes, plays a role in cell survival and multiplication [71]. Current research has also hypothesized that Creb is involved in the metastasis of cancerous cells, which means that its level is significantly increased in people suffering from BC [72]. Cyclin D1 plays a crucial role in the continuation of the cell cycle (Figure 3). To synthesize DNA, Cyclin D1 is required in a significant amount during the gap phase [73]. It is also necessary in the G2 phase for the continuation of the cell cycle [74]. It has been found that the phosphorylation process of Creb is compromised when treating BC cells with PD. So, the tumor-suppressing effect of PD appears to be due to its interference with Creb phosphorylation, which puts a lid on Cyclin D1 and thus terminates the cell cycle [75] (Figure 3).

**Figure 4:**
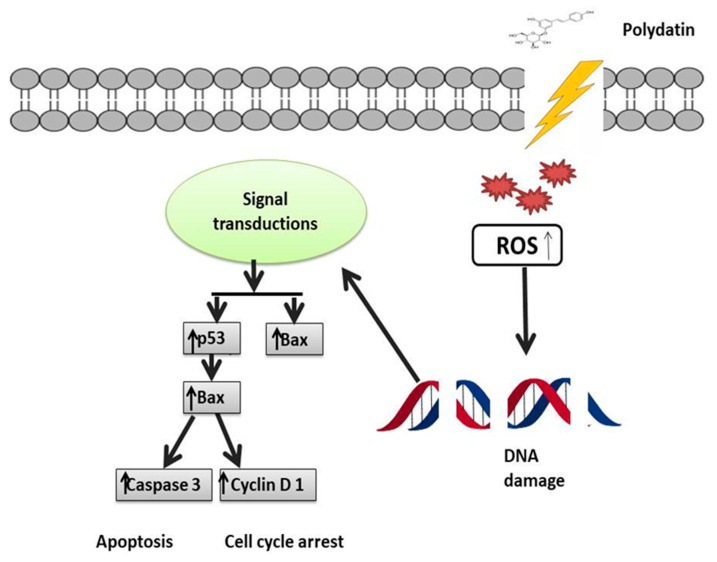
Mechanistic illustration of polydatin activity in the treatment of breast cancer through p53 activation. Activation of p53 leads to activation of p21 and Bax, which in turn leads to cell cycle arrest and apoptosis. ↑ Upregulation, ↓ Downregulation.

Another major factor responsible for breast cancer is the matrix metalloproteinase (MMP) as it disrupts ECM. MMP is responsible for blood supply to cancerous cells, and its activity is modulated via NF-kB [76]. MMP-2 and MMP-9 are known to disturb the extracellular matrix and also play a significant role in metastasis [77]. Moreover, a direct relationship has been found between the levels of vascular endothelial growth factor (VEGF) and the development of cancer. PD is known to counteract all these factors contributing to BC [78,79], as shown in Table 2. Zhang et al. [80] investigated the anticancer activity of PD on the breast cancer cell lines 4T1 and MCF-7 and observed that, compared to the control group, PD at 100 μmol/L substantially suppressed cell growth and migration. Rising levels of the autocrine vascular VEGF are seen as a characteristic of cancer invasion in vitro. The results of Zhang et al. [80] showed that, as compared to the control, PD along with 2-Deoxy-D-glucose significantly suppressed MMP9, MMP2, and VEGF expression. Another major regulator of tumor progression is programmed cell death and apoptosis, which is utilized as a target for BC. Certain caspases like caspase-3,9 and apoptosis-related proteins such as Bcl-2 and Bax should be targeted [80,81]. Research has confirmed that by regulating proapoptotic and antiapoptotic proteins, PD causes cancer cell death [80]. Mitochondrial dysfunction and ROS production are also responsible for malignancy. ROS production is most commonly seen in triple-negative breast cancer (TNBC), thus it can be used as a target in the treatment of TNBC [82]. Certain signaling pathways which are oncogenic are upregulated by the overproduction of ROS like NF-kB, Wnt, MMPs, and EGFR [83,84,85]. ROS are involved in the upregulation of the PI3K/Akt pathway, which ultimately leads to prosurvival signaling. PD also acts as a free radical scavenger [86]. PD treatment thus balances the levels of free radicals within the body and also blocks the prosurvival signaling pathway [87,88] (Figure 4). The energy-making process of cancerous cells is aerobic glycolysis, which is required for their proliferation and migration [89]. To fulfill their energy demands, cancer cells require high levels of glucose, so glycolysis is one of the novel emerging targets of PD [90,91]. Hexokinase 2 (HK2) is an enzyme markedly expressed in cancerous cell glycolysis and which is targeted by PD [92]; PD decreases the levels of this enzyme. Uncontrolled expression of the hypoxia-inducible factor 1α (HIF1α) is also a hallmark of cancer which prevents apoptosis. HIF1α was also found to be impaired by PD [93,94,95]. In one study, the anticancer activity of PD was observed by giving it with D-glucose, using MCF-7, 4T1 cell lines. It was found that proliferation and metastasis was reduced. It was also demonstrated that its antioxidant activity was a major contributor in the treatment of BC because of its ability to reduce ROS, and also by targeting PI3K/AKt pathway that is linked with ROS production [80] (Figure 3).

### 4.5. Cervical Cancer

**Figure 5:**
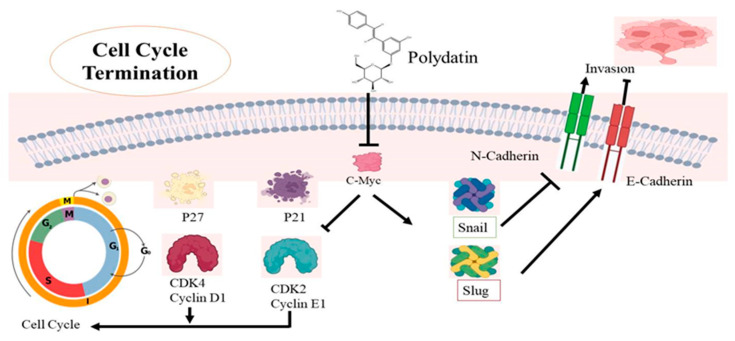
The mechanistic approach of polydatin (PD) activity in terminating cell cycle in cervical cancer cells. PD causes the downregulation of the *c-Myc* gene, which alters two mechanisms involved in cervical cancer. PD causes upregulation of *p21* and *p27*, and also induces repression of *CDK4* and *cyclin D1* as well as *CDK2* and *cyclin E1*; their dysregulation ultimately terminates the G0/G1 phase of the cell cycle. In the second pathway, PD causes Snail and Slug expressions, leading to upregulation of E-cadherin expression and downregulation of N-cadherin, and thus impairing cell metastasis in cervical cancer.

The fourth major reason for death among women is cervical cancer, which is more prevalent in developed countries [96]. Research has revealed that cervical cancer has a link to human papillomavirus (HPV). Smoking, HPV, early sexual activity, and genetic modifications lead to cervical cancer [97,98]. Undoubtedly, treatment options are available, but their outcomes are still uncertain, and there is a need to find other therapeutic alternatives [99,100,101]. PD is known to have anticancer potential and it can target some major factors involved in cervical cancer development [102,103,104]. The cell cycle is under the control of CDKs and cyclins, whose dysregulation leads to uncontrolled cellular multiplication [76]. Research has confirmed that PD causes cell cycle termination at the G0/G1 phase, upregulation of p21 and p27, and also induces repression of CDK4 and cyclin D1 [105]. One major factor affecting cell movement and invasion is EMT, which is controlled by several signaling pathways like NF-kB, MAPK, and other transcription factors [76]. During EMT, structural changes are seen in epithelial cells whose polarity is lost. Some proteins are linked with EMT, from which some are overexpressed and some are downregulated, leading to metastasis. The expression of these proteins is actually targeted by PD to prevent cell invasion, as shown in Table 2. PD causes upregulation of E-cadherin expression and downregulation of Snail and Slug expressions, thus impairing cell metastasis in cervical cancer as the switch from E-cadherin to N-cadherin, which plays a major role in the invasiveness of cancer [105,106,107]. Snail and Slug expressions also play a major role in cancer metastasis [108]. It is well-known that proto-oncogenes are involved in all the major types of cancers. The *c-Myc* gene is one of these specific genes which has been identified in cervical cancer. Along with cyclins and CDKs, the cell cycle is also affected by the expression of the *c-Myc* gene [109] so that its expression can be targeted in treating cervical cancer. *C-Myc* overexpression is seen as a sign of cervical cancer [110]. *C-Myc* gene underexpression has been reported in CaSki and C33A cells after PD exposure, suggesting its possible use for ameliorating cervical cancer treatment [111,112]. A lot of proteins regulate the cell cycle whose expression is controlled by *c-Myc* by altering signaling pathways [113,114]. Downregulation of the *c-Myc* gene by PD will impair the overexpression of these proteins. Actually, the mechanism behind the regulation of the cell cycle by *c-Myc* is responsible for the downregulation of the expression of both *p21* and *p27* [115,116]. Both are tumor-suppressant genes that arrest the cell cycle during the gap and the synthesis phases. It is known that a particular CDK interacts with a particular cyclin, allowing the continuation of the cell cycle [117]. The CDK4-Cyclin D1 interaction causes cell proliferation but p21 has the ability to stop the cell cycle by preventing this association [118] (Figure 5). The same effect is seen with p21 and p27 and CDK2-Cyclin E1 combinations, CDK2-Cyclin E1 also being a major contributor to cell cycle progress. PD was found to downregulate both p21 and p27 in cervical cancerous cells, thus stopping cell cycle progression [119]. The *c-Myc* gene also increases Snail and Slug expression, thus inhibiting N-cadherin, promoting E-cadherin, and leading to the prevention of the survival of cancer cells [120] (Figure 5). The anticancer potential of PD was observed on HeLa cell lines, and it was found that this stilbene decreases mRNA and protein expression levels of PI3K, AKT, mTOR, leading to apoptosis. It also causes cell death in cervical cancer by targeting the ROS/PI3K/AKT/mTOR pathway [121]. Another study was conducted by using female nude mice. PD (100 mg/kg) was given by injection and the results showed that the tumor size was small and its progression was also reduced [105] (Figure 5).

**Table 2:** Anticancer activity of polydatin on different types of cancer.

Cancer Type	Cell Line	Type of Study	Concentrations of PD	Molecular Targets	Mechanism of Action	References
Breast cancer	MDA-MB-231 MCF-7	In vitro	2, 4, 6 µM	↑ p38 ↑ JUN ↑ ERK ↑ AKT	Promotes apoptosis by MAPK/ERK & P13K/AKT pathways	[122]
	4T1 MCF-7	In vitro	5.53 mmol/L 8.67 mmol/L	↓ p-PI3K/PI3K ↓ p-AKT/AKT	Inhibits P13K/AKT pathways	[80]
Cervical cancer	CaSki C33A	In vitro	0.1, 10,100, 500 µM	↑ p21 ↑ p27 ↓ Cdk4 ↓ Cdk2 Cyclin D1 ↓ Cyclin E1	Inhibits growth promoter proteins and cell cycle arrest	[105]
	HeLa	In vitro	50, 100, 150 μmol/L	↓ PI3K ↓ AKT ↓ mTOR P70S6K ↓ c-Myc	Induced apoptosis by suppression of PI3K/AKT/mTOR signaling	[15]
Lung cancer	A549 NCI-H1975	In vitro	6 µ mol/L	↓ Bcl 2 ↑ Bax ↑ Cyclin D1	Cell cycle arrest and apoptotic pathway	[123]
	A549 and H1299 cells	In vitro		↓ NLRP3 ↓ ASC ↑ pro-caspase-1 ↑ NF-kB ↑ p56	Promotes apoptosis and *NLRP3 inflammasome inhibition by NF-kB*	[20]
Ovarian cancer	OVCAR-3, A2780, and HO-8910	In vitro	50 μM	↑ P13K ↑ AKT	AKT signaling	[124]
	SKOV-3 and OVCAR-8	In vitro	5, 10, 50, 100 μM	↓ Her-2 ↓ EGFR ↓ VEGF ↑ ERK ↑ PARP-1	Down/upregulation of various cell signaling molecules	[125]
Liver cancer	HCC cells	In vitro	100 μM 150 μM	↓G2/M Phase ↓ STAT3 ↓ AKT ↓ JAK1	Cell cycle arrest JAK1/STAT3 and P13K/AKT signaling	[53]
	HepG2 SMMC-7721	In vitro	1, 3, 10, 30, and 100 µM	↓ β-catenin ↓ Bcl 2 ↑ Bax ↑ Caspase-3 ↑ Caspase-9	Apoptotic pathway	[80]
	HepG2	In Vitro	(10, 30, and 100 μM)	↓ Bcl 2 ↑ Bax ↓ Wnt	Wnt signaling Apoptotic pathway	[54]
Colon carcinoma	CaCo-2	In vitro	1–50 μM	↓ DNA synthesis ↓G0/G1	Cell cycle arrest	[56]
	Caco-2	In vitro	100 240 μM	↓ AKT ↑ PARP ↓ Erk-1 ↓ Erk-2	Regulation of Akt/PKB signaling	[55]
Human myeloma cells	RPMI 8226	In vitro	50, 100, 200 μmol/L	↑ Caspase-3 ↑ Caspase-9 ↑ Bax ↓ mTOR/p70s6k	Apoptotic pathway	[58]
Osteosarcoma cells	143B MG63	In vitro	10, 30, 100 μM	↑ Caspase -3 ↓ Bcl 2 ↑ Bax ↓ β-catenin	Regulation of Apoptotic pathway	[63]
Lukemia cells	MOLT-4	In vitro	1, 4 or 20 µM	↓ Cyclin D1 ↓ CYCLIN B1 ↓ Bcl2	Cell cycle arrest and apoptotic pathway	[126]
Nasal carcinoma	CNE	In vitro	5, 10, 20 µM	↓ AKT ↑ Endoplasmic ↑ Reticulum stress ↑ Caspase 3 ↑ Caspase 4 ↑ Caspase 9	Regulation of apoptotic pathway molecules	[127]
Laryngeal cancer	AMC-HN-8 cells	In vitro	2, 4, 6 µM	↓ PDGF-B ↓ Ki67 ↓ Bcl 2 ↑ Bax ↓ Akt	Regulation of apoptotic pathway and Akt signaling molecules	[128]

NF-KB: Nuclear Factor kappa-light-chain-enhancer of activated B cells, Wnt: Wingless/Integrated, EGFR: Epidermal growth factor receptor, MMP: Matrix metalloproteinase, VEGF: Vascular endothelial growth factor, ROS: Reactive oxygen species, EMT: Epithelial-to-mesenchymal transition, Bcl-2: B-cell lymphoma 2, Bax: BCL2 associated X apoptosis regulator, NLRP3: NLR family pyrin domain containing 3, PI3K: Phosphatidylinositol 3-kinase, PD: Polydatin, Akt: Serine/threonine kinase 1, mTOR: Mammalian target of rapamycin, ERK: Extracellular signal-regulated kinase, PARP: poly adenosine diphosphate-ribose polymerase. ↑ Upregulation, ↓ Downregulation.

### 4.6. Lung Cancer

**Figure 6:**
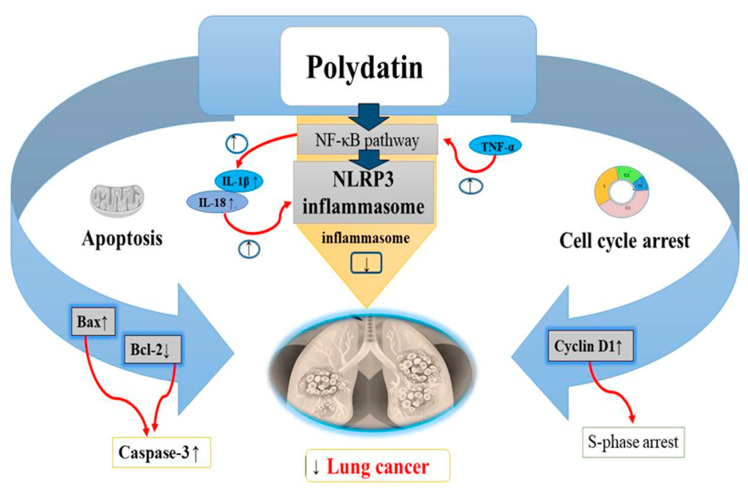
Anticancer effects of polydatin on lung cancer via the apoptotic pathway by increasing BAX levels, decreasing Bcl-2 levels and increasing caspase-3 levels, induction of cell cycle arrest at S phase by decreasing the cyclin D1 levels, and inhibition of the NLRP3 inflammasome by suppressing the NF-kB pathway in tumor cells. Upregulation ↑ Downregulation ↓.

Lung cancer, a growing health problem worldwide was found to be the most common type of cancer compared to other cancers [129]. Its prevalence and increase in mortality are strongly related to the history of smoking [130]. Many treatments such as targeted chemotherapies, radiotherapies, and surgery are used to cure lung cancer but despite advancements in these therapies, lung cancer still remains antagonistic in nature with poor survival rate. Chemotherapy is used recurrently against lung cancer in progressive stages but with deleterious consequences for patients [131]. Lung cancer cells treated with doses till 6 μM PD, showed a dose-dependent reduction in Bcl-2 and cyclin D1 levels as well as an increase in Bax, leading to cell cycle arrest at the S phase. Interestingly, the human non-cancerous nasopharyngeal cell line exhibited lower cytotoxicity when exposed to PD. This was also corroborated by several researchers [132]. A recent study found that PD is advantageous for lung cancer inhibition [132]. The initiation of apoptosis in lung cancer cells is considered a good anticancer target [133,134]. In cancer cells, the antiapoptotic protein Bcl-2 is not capable of forming heterodimeric complexes with the proapoptotic protein Bax, resulting in high Bax levels. Increased Bax/Bcl-2 ratios upregulate the release of cytochrome C from mitochondria into the cytosol, leading to caspase-3 stimulation and apoptosis activation [135,136]. Research has revealed that 6 μmol/L of PD activates apoptosis in A549 lung cancer cell lines by impairing Bcl-2 levels and upregulating Bax levels [132] (Table 2). On the other hand, cell cycle arrest of the cancer cells is considered to be a potential target against cancer progression [137,138] (Figure 6). Cyclin D1 expression should be high for the normal initiation of DNA synthesis, while cyclin D1 levels should be low during the S phase [139]. Overincrease in cyclin D1 expression has been reported in many cancers including lung cancer [140,141]. In recent in vitro studies, PD has been found to hamper lung cancer cell (A549 and NCI-H1975 cells) progression by decreasing cyclin D1 levels and arresting cells at the S phase [132] (Table 2). PD also exerts some antioxidant and anti-inflammatory activities by inhibiting secretion of inflammatory oxidative factors or by increasing the scavenging of free oxygen radicals [142]. In recent studies, NLRP3 (NLR family pyrin domain containing protein 3) inflammasome has been observed to take part in inflammation related to cancer and tumor progression. Thus, suppression of the NLRP3 inflammasome might also be an effective strategy in the treatment of lung cancer [143]. NF-κB pathway has been revealed to be the significant marker in NLRP3 inflammasome activation. Increased levels of TNF-α activates the NF-κB pathway which then upregulates the IL-1β and IL-18 levels, causing the activation of NLRP3 inflammasome. PD (50 μM) has been shown to attenuate the multiplication and metastasis of human A549 and H1299 cell lines through suppression of the NLRP3 inflammasome by suppression of the NF-κB pathway [20] (Figure 6).

### 4.7. Ovarian Cancer

**Figure 7:**
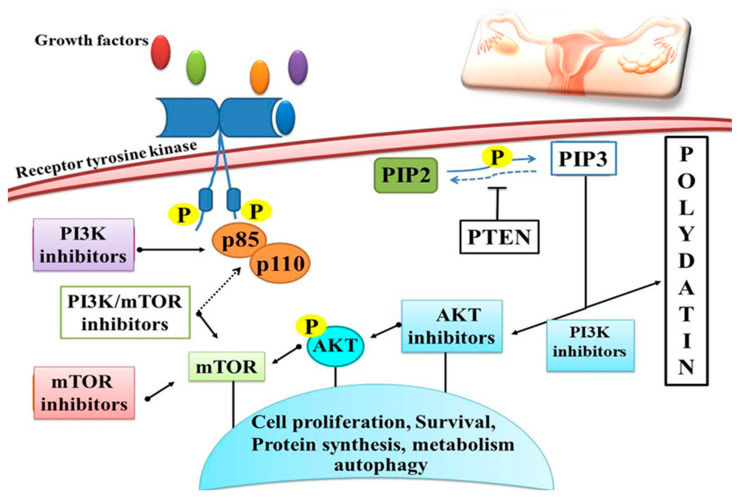
Schematic overview of polydatin activity on the PI3K/AKT/mTOR signaling pathway with different strategies for inhibition. PD induces apoptosis in cancer cells through the PI3K/Akt/mTOR signaling pathway and protects against inflammatory damage, as well as inhibiting cell proliferation, survival and protein synthesis through protein phosphorylation.

Ovarian cancer is the most frequent cause of death among women within gynecological cancers worldwide [144]. Researchers have discovered in recent years that Chinese medicine displays a significant anticancer activity with fewer side effects as compared to synthetic drugs. PD ought to enhance ovarian cancer cell susceptibility to radiations, limit cell growth, and promote apoptosis. It has been found that PD has the potential to facilitate cancer cell apoptosis [145] (Table 2). PI3K signaling controls cell growth, death, and survival [146]. PD triggered apoptosis in cancer cells, namely ovarian cancer cells, and protected against inflammatory damage through the phosphoinositide, 3-kinase/protein kinase B/mammalian target of rapamycin (mTOR) pathway [75,147]. PD was able to successfully limit the growth of the ovarian cancer cell lines OVCAR-3, A2780 and HO-8910. There was a decrease in proliferation, migration and invasion after treatment with PD in the cancer cell lines OVCAR-3, A2780, and HO-8910 [124]. In addition, PD inhibited PI3K, which in turn increased extracellular signaling and regulated ERK phosphorylation, thus inhibiting cancer cell growth [124] (Figure 7). The anticancer effect of PD was demonstrated by the downregulation of tumor suppressor genes via inhibition of the PI3K/Akt signaling and upregulation of bone morphogenetic protein 7 (BMP7) [127]. Inhibiting the proliferation, migration, and invasion of ovarian cancer cells is one of the main PD’s effects [148]. PD prevents ovarian cancer cell proliferation, migration and invasion by inhibiting the expression of the PI3K protein, which is the cornerstone of ovarian cancer treatment. By decreasing EGFR phosphorylation and production of ERK and VEGF, PD inhibited the cellular aggregation of ovarian cancer cell lines in three dimensions. At concentrations of 5–100 μM, PD was shown to suppress growth of the ovarian cancer cell lines SKOV-3 and OVCAR-8 by decreasing EGFR phosphorylation levels, which in turn increases the likelihood of the cells committing suicide. [149]. Earlier investigations utilising polydatin have suggested that this compound suppresses PI3K protein expression and blocks growth, migration and invasion of OVCAR-3, A2780 and HO-8910 cells. It appears that PI3K is the target of PD, since increasing PI3K protein expression greatly attenuates the inhibitory impact of PD on proliferation, migration and invasiveness of OVCAR-3, A2780 and HO-8910 cell lines. Experimental evidence supports the possible use of PD in the treatment of ovarian cancers because of its ability to suppress the growth, migration, and invasion of these cell lines by downregulating PI3K protein expression [124].

PD inhibited OVCAR-8 and SKOV-3 cell growth in a dose-dependent manner. A growth rate decrease was achieved by triggering apoptosis through the cleavage of poly(ADP-ribose) polymerase (PARP-1) at PD concentrations of 50 and 100 µM. In the SKOV-3 line, PD inhibited Her-2 and EGFR phosphorylation and Erk expression, as well as the VEGF, when used at greater dosages, and stimulated Erk activation in the OVCAR-8 cell line. Results of this investigation showed that PD has the potential to block the formation of 3D cell aggregates in ovarian cancer cell lines by influencing a variety of signaling molecules. However, more experiments for the in vivo testing of resveratrol and PD are needed to determine their potential therapeutic values [125]. Several other cancer cell lines were shown to be significantly inhibited by PD (Table 2).

## 5. Underlying Polydatin Anticancer Mechanisms of Action

PD has been studied extensively for its potential as a chemopreventive agent and chemotherapeutic treatment for halting or reversing carcinogenesis at multiple stages. PD, like other phytochemicals, can act as a suppressive agent on multiple impaired signaling pathways; as such, it has been classified as a functionally pleiotropic agent, capable of expressing its activity on multiple targets in cancer cells while causing only mild side effects in healthy cells. Important cellular changes include increased oxidative stress, overproduction of growth-regulatory hormones, accelerated transition of cells through cell cycle checkpoints, abnormal cell proliferation, genome instability, abnormal response to signals or other stimulators of programmed cell death, uncontrolled neoangiogenesis, and altered host immune responses. In addition, oxidative stress-related damage (including DNA damage, protein oxidation, and lipid peroxidation) is mitigated by antioxidant, anti-inflammatory, and immunomodulatory activities, which also boost immune oncosurveillance [150]. PD inhibits the monooxygenase cytochrome P450 isoenzyme CYP1 A1, the enzyme deputed to the liver metabolism of xenobiotics, and hence it can also function as a cancer-blocking agent by preventing the transformation of procarcinogens into carcinogens [151].

One of the primary functions of phytochemicals is to inhibit growth-signaling activity. A transmembrane tyrosine kinase is activated by ligands; the epidermal growth factor (EGF), and its related receptor (EGF-R) represent two of the resveratrol’s primary targets. Overexpression of EGF-R is a hallmark of malignant tumors with aggressive characteristics because it stimulates cell growth and proliferation [152]. Resveratrol, acetyl-resveratrol and polydatin showed dose-dependent antigrowth activities against 3D cell aggregates of EGF-R/Her-2-positive and -negative ovarian cancer cell lines [125]. The phosphorylation of Her-2 and EGF-R, as well as the expression of extracellular-signal-regulated kinases (ERK) and VEGF, were all significantly reduced when resveratrol, PD and acetyl-resveratrol were tested at high concentrations on the positive ovarian cell line [125].

**Figure 8:**
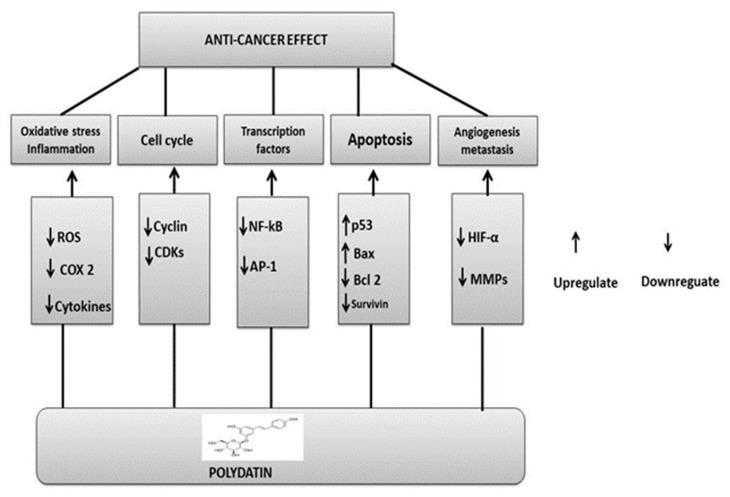
Polydatin anticancer mechanisms: upregulation or downregulation of various pathways.

PD and its analogues displayed an interactive effect with TRAIL (tumor necrosis factor-related apoptosis-inducing ligand) and triggered apoptosis (programmed cell death). Particularly resistant to TRAIL are androgen-dependent LNCaP cells in prostate cancer; however, PD downregulated the PI3K/AKT pathway to make these cells more responsive to TRAIL-mediated apoptosis. Treatment of LNCaP cells with PD induced ROS production, mitochondrial membrane potential decreases, and translocation of the Bcl2-like protein 4, also known as Bcl-2-associated X protein (Bax), and p53 tumor suppressor protein. Proteins such as cytochrome c CASP-3 and CASP-9, apoptosis-inducing factor (AIF), the second mitochondria-derived activator of caspase/direct inhibitor of apoptosis-binding protein with low pI (Smac/DIABLO), and protein high-temperature requirement serine protease A2 (HtrA2), also known as Omi, are among the proapoptotic proteins released by mitochondria in relation to PD analogues [153] (Figure 8).

## 6. Concluding Remarks

Currently, phytomedicine is gaining the attention of researchers and nutritionists due to its diversified pharmacological activities. The data collected showed the anticancer activities of PD. So, it would be valuable to investigate the in-depth mechanisms of PD activities. In recent years, PD has gained attention as a promising anticancer drug due to its potential to modulate many signaling pathways associated with cancer development. PD’s anticancer effects arise from the fact that it boosts antioxidant activity. Due to its long-conjugated chemical structure, this molecule displays powerful antioxidant properties. PD may trigger apoptosis by raising the Bax/Bcl-2 ratio and decreasing Wnt/catenin signaling to kill cancer cells. It also targets cell cycle arrest to inhibit the development of BC. Among the several oncogenic pathways involved in BC the cell cycle arrest is linked to PD’s apparent interference with Creb phosphorylation, which in turn downregulates Cyclin D1. PD therapy also restores the body’s natural equilibrium of free radicals. Tumor size and rate of cancer progression were both significantly decreased after intravenous administration of PD. Furthermore, it was shown that PI3K levels are decreased in PD-treated patients. PD also protects against inflammatory damage and inhibits cell proliferation, survival, and protein synthesis by targeting the PI3K/Akt/mTOR signaling pathway, which is activated in cancer cells. Liver xenobiotic metabolism is mostly carried out by the monooxygenase cytochrome P450 isoenzyme CYP1 A1, which is inhibited by PD. As a blocking agent, it can stop procarcinogens from becoming carcinogenic. In this review, we have thus emphasized the anticancer activity of PD and its underlying pharmacological modes of action. The anticancer effect of PD should also be investigated through thorough preclinical trials. There is thus a need for carefully monitored human studies to determine its therapeutic efficacy.

## Figures and Tables

**Table 1:** Polydatin contents in vegetable and fruits samples.

Plant	Plant Organ	Solvent for Extraction	Polydatin Quantity (µg/100 g)	References
Banana	Fruit	Methanol	1.70 µg/100g	[35]
Lychee	Fruit	Methanol	1.00 µg/100g	[35]
Pomegranate	Fruit	Methanol	7.56 µg/100g	[35]
Waterchestnut	Fruit	Methanol	0.50 µg/100g	[35]
Waxapple	Fruit	Methanol	1.58 µg/100g	[35]
Tomato	Fruit	Methanol	4.22 µg/100g	[35]
Chili pepper	Fruit	Methanol	14.47 µg/100g	[35]
Bell pepper	Fruit	Methanol	36.22 µg/100g	[35]
Grape	Fruit	Methanol	71.54 µg/100g	[35]
Gauva	Fruit	Methanol	0.72 µg/100g	[35]
Lemon	Fruit	Methanol	17.00 µg/100g	[35]
Strawberry	Fruit	Methanol	100 µg/100g	[35]
Orange	Fruit	Methanol	5.31 µg/100g	[35]
Mushroom	Fruit	Methanol	2.16 µg/100g	[35]
Pear	Fruit	Methanol	13.10 µg/100g	[35]
Soya bean	Fruit	Methanol	42.58 µg/100g	[35]
Gallic bulb	Fruit	Methanol	2.00 µg/100g	[35]
Chrysanthemum	Fruit	Methanol	5.20 µg/100g	[35]
Coca	Fruit	Methanol	7.56 µg/100g	[35]
White dammar	Leaves	Diethly ether	0.22 mg/g	[38]
Peanut	Seeds	Ethanol	0..128 µg/100g	[39]
Cocoa	Seeds	Hexane	7.14 µg/g	[40]
Norway spruce	Phloem	Methanol	16 mg/g	[23]
Norway spruce	Bark	Methanol	1.3 mg/g	[23]
